# Anesthetic Considerations for Cesarean Delivery After Uterine Transplant

**DOI:** 10.7759/cureus.13920

**Published:** 2021-03-16

**Authors:** Islam Mohammad Shehata, Sabri Barsoumv, Amir Elhass, Giustino Varrassi, Antonella Paladini, Dariusz Myrcik, Ivan Urits, Alan D Kaye, Omar Viswanath

**Affiliations:** 1 Department of Anesthesiology, Ain Shams University, Cairo, EGY; 2 Department of Anesthesiology, Cleveland Clinic, Cleveland, USA; 3 Department of Neurosurgery, Desert Regional Medical Center, Palm Springs, USA; 4 Research, Paolo Procacci Foundation, Rome, ITA; 5 Department of Life, Health and Environmental Sciences, University of L'Aquila, L'Aquila, ITA; 6 Emergency, Medical University of Silesia, Bytom, POL; 7 Department of Anesthesia, Critical Care and Pain Medicine, Beth Israel Deaconess Medical Center, Harvard Medical School, Boston, USA; 8 Department of Anesthesiology, Louisiana State University Shreveport, Shreveport, USA; 9 Pain Management, University of Arizona, Phoenix, USA

**Keywords:** uterine transplant, donor uterus, anesthesia, pregnancy, mayer-rokitansky-küster-hauser syndrome, postoperative pain, cesarean delivery

## Abstract

Infertility has many etiologies and can have devastating consequences for young couples attempting to bring children into the world. Uterine factor for infertility is related to either uterine agenesis (Mayer-Rokitansky-Küster-Hauser [MRKH] syndrome), unexpected hysterectomy, or presence of a nonfunctioning uterus. In this review, a patient with MRKH syndrome underwent donor uterus transplantation at the Cleveland Clinic, conceived, and delivered the first healthy baby in the United States and the second worldwide. Additionally, we review the pertinent literature on anesthesia problems. Donor-related uterine transplant is a recent medical innovation requiring multidisciplinary expertise. In patients who deliver successfully, according to the current literature, the transplanted uterus can be used for one more pregnancy only if the mother so desires, otherwise cesarean hysterectomy (C-Hyst) should be performed. In the observed case, C-Hyst was performed because the patient developed placenta accreta and the couple desired no further pregnancy. In summary, with our limited data, careful management of these patients is required to ensure the best outcome for the mother and the newborn fetus.

## Introduction and background

In June 2018, the first birth from a deceased donor uterine transplant (UTx) was carried out successfully at the Cleveland Clinic in the United States. It was the second such birth worldwide. The procedure was made possible by a team including specialists ranging from transplant surgeons to social workers. The transplant and birth are part of an ongoing clinical trial offering hope to women unable to have children related to a uterine factor infertility known as the Mayer-Rokitansky-Küster-Hauser (MRKH) syndrome.

The successful UTx was performed at the Cleveland Clinic in February 2017. The cesarean hysterectomy (C-Hyst) proceeded uneventfully. The mother and the baby did exceptionally well. The mother was transferred to the post-partum floor after a brief stay in the post-anesthesia care unit. The baby who was born at 36 weeks was kept in the neonatal intensive care unit for two weeks before discharge home.

To date, few, if any, publications in the medical literature have discussed anesthesia considerations of the parturient after solid organ transplantation (e.g., heart, kidney, and liver). In this regard, there are no articles discussing anesthesia considerations for UTx. Therefore, this case gives the opportunity to describe the history and perioperative events of a patient who underwent UTx and delivered a healthy baby.

## Review

History of uterine transplant

UTx has proven to be a relatively new treatment for women diagnosed with absolute uterine factor infertility (AUFI). AUFI is attributed to either uterine agenesis (MRKH syndrome), unexpected hysterectomy, or the presence of a nonfunctioning uterus. A survey involving 40 women diagnosed with AUFI demonstrated that 39 of them would undergo UTx. Moreover, in the majority of countries worldwide, gestational surrogacy is not practiced based on legal, ethical, or religious concerns, which reflects significant interest in the public for UTx [1].

The success of UTx is evaluated by the delivery of a healthy baby as evidence of graft function. Therefore, although the first clinical UTx was in 2000, the first baby born after successful UTx was in Sweden in 2014 [2]. Inspired by this success, approximately 42 UTx procedures have now been reported globally, and at least 12 live births have been reported in recent years [3]. The improved outcome increases the number of such cases which challenges the anesthesia team. Thus, in this review, we aim to address important perioperative anesthetic considerations.

Physiology of transplanted uterus

Recently, it has been appreciated that the autonomic innervation regulates physiological uterine functions and changes according to the blood level of sex hormones during different phases of pregnancy. However, the transplanted, denervated uterus responds qualitatively to hormones as does the native uterus with similar systemic physiological changes during pregnancy [4].

From an anesthesia standpoint, the reported cases have not depicted any change in uterine behavior to the commonly used intraoperative ecbolic therapy (hormonal nature) during cesarean delivery. Moreover, it has been described that the uterus contracts well, especially with intravenous oxytocin [5,6].

Pregnancy after solid organ transplantation is associated with an increased risk of complications, including pre-eclampsia (22%) and preterm birth (46%), especially after uterine transplantation [7]. Therefore, it is imperative to consider such cases as high-risk pregnancy, which should be managed by a multidisciplinary team that includes transplant surgeons, obstetricians, neonatologists, obstetric specialized intensivists, and anesthesiologists.

The denervated uterus shows less resistant blood vessels related to the absence of autonomic-mediated vasoconstrictive mechanisms, which theoretically should reduce the risk of pre-eclampsia. However, the coincident pre-eclampsia is attributed to several plausible factors such as immunosuppressive therapy, old age of the transplanted uterus, and in-vitro fertilization which has been involved in such cases [8,9].

Preoperative evaluation

Timing of Delivery

There is no consensus on the optimal transplant-embryo transfer interval after UTx. However, in 2005, the American Society of Transplantation Consensus recommended conception one year after transplant as long as graft function is stable [10].

Most cases after UTx have waited for one year to ensure healing and stabilization from the immunosuppressive therapy. However, there is a tendency in recent reported cases to shorten the time to seven months. Review of previous cases has found that period adequate to reach optimum endometrial thickening.

The Brännström recommendation is to deliver at 35-36 weeks to avoid potential hazards to fetal vitality beyond that time. Therefore, the optimal time to delivery should be around 15-20 months after transplant surgery [5,6].

Immunosuppressive Therapy

The uterus is not a vital organ with a direct effect on the metabolic profile of the parturient. Therefore, there is no specific allograft function to be evaluated such as for solid organ transplantation.

However, comprehensive preoperative anesthetic management in these parturients must evaluate essential considerations. The most important consideration is immunosuppressive therapy, its complications, especially hematological effects, its interaction with anesthetic drugs, and the potential risk of infection [11].

Several regimens of immunosuppression have been modified over the years either for induction or maintenance therapy. The recent trend has shifted toward reduction of steroids by implementation of different drugs (Table 1) [12].

**Table 1:** Common drugs utilized in patients receiving uterine transplantation. NRATG = Nashville rabbit anti-thymocyte globulin; NRATS = Nashville rabbit anti-thymocyte serum; ALG = anti-lymphocyte globulin; TOR = target of rapamycin

General names	Generic names
Corticosteroids	Prednisone, methylprednisolone
Calcineurin inhibitors	Tacrolimus (or FK-506), cyclosporin (or cyclosporin A)
Antimetabolites	Azathioprine, cyclophosphamide, mycophenolate mofetil, mycophenolate sodium
Polyclonal antibodies	Antithymocyte globulin (equine), NRATG, NRATS, ALG
Anti-CD3 monoclonal antibodies	Muromonab-CD3
Anti-CD52 monoclonal antibodies	Alemtuzmab
Anti-IL-2 receptor monoclonal antibodies	Basiliximab
TOR inhibitors (or rapamycin)	Daclizumab, sirolimus

Pregnancy is associated with significant increase in blood volume by 40% and cardiac output by 30%, which alter immunosuppressive drug clearance. Therefore, the dose should be closely monitored by serum levels to avoid either subtherapeutic levels, which aggravate the risk of rejection, and supratherapeutic levels, which can lead to infection and/or other significant side effects [13].

Immunosuppressive maintenance therapy is the hallmark of preoperative assessment related to its recognized adverse effects, which may alter the choice of the anesthetic plan (Table 2) [14].

**Table 2:** Immunosuppressive maintenance therapy common adverse effects. CyA = cyclosporine A; Tacr = tacrolimus (FK506); Aza = azathioprine; Ster = steroids; MMF = mycophenolate mofetil; ATG = anti-thymocyte globulin; OKT3 = monoclonal antibodies directed against CD-3 antigen of the surface of human t-lymphocytes

	CyA	Tacr	Aza	Ster	MMF	ATG	OKT3
Anemia	-	-	+	-	+	-	-
Leucopenia	-	-	+	-	+	+	+
Thrombocytopenia	-	-	+	-	+	-	-
Hypertension	++	+	-	+	-	-	-
Diabetes	+	++	-	++	-	-	-
Neurotoxicity	+	+	-	+	-	-	-
Renal insufficiency	+	++	-	-	-	-	-
Anaphylaxis	-	-	-	-	-	+	+
Fever	-	-	-	-	-	+	+

To date, few studies have investigated how immunosuppressive drugs may alter the pharmacological actions of anesthetic drugs. With regards to intravenous anesthesia, cyclosporine enhances barbiturate anesthesia and fentanyl analgesia in mice; however, the precise mechanism(s) of these interactions remain unclear [15]. Moreover, both cyclosporine and tacrolimus increase blood levels of benzodiazepines, which may require dose adjustment [16].

Most muscle relaxant have no significant interactions; however, enhancing effects of cyclosporine can result in prolongation of neuromuscular blockade. The effect is evident, especially after vecuronium, atracurium, and pancuronium, which mandates decreasing the dose and expecting longer recovery time [17].

The dose of immunosuppressive drugs should be maintained perioperatively with consideration of stress dosage of steroids in parturients who have been withdrawn from steroid therapy and those with refractory hypotension [18].

Opportunistic Infection

Surveillance for opportunistic infection with a high index of suspicion should be an integral part of preoperative evaluation. The high prevalence of infection in these patients is attributed to the immunosuppressive therapy and immunomodulation effects of pregnancy [19].

Anesthetic management

Surgical Consideration

Cesarean delivery is undertaken through a midline incision and not through the conventional Pfannenstiel incision for better visualization. The midline incision is also indicated because of potential removal of the transplanted uterus and to avoid lifelong immunosuppression. C-Hyst after transplanted uterus is a complex challenge. It necessitates a multidisciplinary team of obstetricians, transplant surgeons, and vascular surgeons. Availability of adequate amounts of leukocyte-poor irradiated blood products is a requirement related to expected bleeding. The risk of bleeding is attributed to adhesions following the transplant surgery and hematological complications of immunosuppressive drugs such as anemia and thrombocytopenia. Moreover, vasoplegic uterine vessels can increase bleeding [5,6].

Choice of Anesthesia Technique

The choice of the anesthetic technique for cesarean delivery should be personalized based on the anesthetic judgement of maternal and fetal risk factors and patient preference. Regional anesthesia techniques are favored in comparison to general anesthesia in cesarean delivery [20]. Finally, planned C-Hyst following cesarean delivery makes the choice of anesthesia more problematic. There are advantages and disadvantages to both regional and general anesthesia techniques and, at present, there is a paucity of data to define the best practice strategy. In this regard, secondary analysis of Maternal-Fetal Medicine Units of Caesarean registry database [21] showed that composite maternal and neonatal morbidities were similar between the two groups of women who had C-Hyst under general anesthesia and those with planned regional anesthesia [22].

A year after that study, a retrospective cohort study of 129 parturients undergoing cesarean delivery with suspected morbidly adherent placenta from 1997 to 2015 demonstrated that regional anesthesia was delivered successfully to 95% of the cases. Of the 72 parturients requiring hysterectomy given regional anesthesia, only 21% of the cases required conversion to a general anesthesia. It had been reported that converted patients have had a higher rate of packed red blood cell transfusions. The study also assumed the only independent predictor of conversion to general anesthesia was a longer surgical duration. Therefore, they recommended selective conversion to general anesthesia during hysterectomy in these patients focusing on those with the highest levels of surgical complexity [23].

It is imperative to consider that hysterectomy at the end of the cesarean delivery makes UTx the first temporary organ transplant which creates new life from an infertile couple only once in a lifetime, without any possibility to have further pregnancies. Therefore, the moment of seeing the baby delivered has numerous mixed emotions for both the mother and the father [24].

Thus, regional anesthesia as a primary anesthetic technique with backup conversion to general anesthesia in case of prolonged surgery might be the best compromise given all considerations. It allows the infertile mother to be awake for the birth of her precious baby and to enjoy that moment which will not happen again with removal of the uterus.

Further, reviewing 22 studies of the Cochrane Pregnancy and Childbirth Group’s Trials Register showed no significant difference in Apgar scores at five minutes between regional anesthesia and general anesthesia [25]. The same finding was approved for the need of neonatal resuscitation with oxygen. However, regional anesthesia until delivery of the fetus, often pre-term, will minimize neonatal exposure to general anesthesia-related drugs, which can potentially cause neonatal respiratory and central nervous system depression.

The above-mentioned technique provides good postoperative analgesia for the midline incision, especially if an epidural technique is employed. Epidural anesthesia will allow both postoperative continuous epidural infusion technique of local anesthetic and/or opioid and patient-controlled epidural analgesia [26].

To consider epidural anesthesia, a coagulation profile and platelet count should be cautiously checked because of potential side effects related to immunosuppressive therapy. Moreover, strict antisepsis is critical because the UTx recipient parturients are at an increased risk of infections. Epidural abscess is a rare, but catastrophic consequence with increased risk factors in these patients [27].

Anesthesia Choice and the Immune System

Another aspect that should be considered when choosing anesthesia is the immunomodulation effect of anesthesia choice on both innate and adaptive immune systems. The immunomodulation effect is either direct, by affecting the functioning cells, or indirect, by altering the stress response, in a parturient receiving immunosuppressive drug.

There are contradictory data between earlier studies which showed that regional anesthesia preserves immune function better than general anesthesia and recent studies which indicated no significant difference between both groups. Therefore, no technique of anesthesia can be recommended in favor of another in terms of effects on the inflammatory response [28].

The impact of anesthesia includes negative and positive effects. Perioperative immunosuppression would theoretically increase the risk of infection and anti-inflammatory effects would provide benefits in case of systemic and local inflammation. Given these considerations, over recent decades, the immune dysregulation effects of different anesthetics have been described [29]. With regards to general anesthesia agents, propofol, a common induction agent, has been reported to exert anti-inflammatory effects. Propofol causes impairment of monocyte and neutrophil functions, including respiratory burst, chemotaxis, and phagocytosis, which may be attributed to its lipid solvent [30]. On the other hand, fentanyl and all other opioids are known to affect natural killer cell cytotoxicity and cell counts [31]. With regards to volatile anesthesia, there are limited data; however, sevoflurane has been shown to induce a more pronounced suppression of cytokine release than isoflurane or enflurane [32].

Monitoring

Monitoring in the parturient post UTx should be tailored to hemodynamic status, medical complications (e.g., pre-eclampsia), and surgical procedure, which involves C-Hyst in the same session. Strict asepsis should be applied if invasive monitoring is planned related to fluid shifts during the hysterectomy. Ultrasound-guided insertion of different vascular lines should be considered because of increased possibility of vascular complications. The risk is attributed to previous placement of lines during the original transplant surgery and postoperative care period [33].

Postoperative care

Unlike other organ transplant patients, immunosuppressive therapy is discontinued after the hysterectomy which permits breast feeding of the newborn. Postoperative analgesia may be achieved using just nonsteroidal anti-inflammatory drugs in case of moderate pain [34]. In case pain is severe, multimodal pain management should be employed [35], choosing the efficacious combinations [36], but early removal of an epidural catheter may be preferable to avoid the potential risk of infection. Thromboprophylaxis measures are indicated in such patients because of the high risk of thromboembolic complications after cesarean delivery [37].

## Conclusions

Medical science has evolved to now provide patients who otherwise could not carry a fetus to receive uterine transplantation and successfully deliver a newborn fetus. These patients require immunosuppressive agents and other significant considerations throughout pregnancy. After the delivery, the patient undergoes a hysterectomy. Therefore, with limited data, careful management of these patients is required to ensure the best outcome for the mother and the newborn fetus.
